# Testicular mixed germ cell tumour with isolated skip metastasis to unilateral pleura: First case reported in the literature

**DOI:** 10.1016/j.ijscr.2019.08.029

**Published:** 2019-09-04

**Authors:** Manmohan Kamat, Shravani Shetye, Neeraj Pratap Singh, Kartik Nattey, Seema Barman

**Affiliations:** Nanavati Superspeciality Hospital, Mumbai, India

**Keywords:** Testicular germ cell tumour, Mixed germ cell tumour, Metastatic testicular tumour, Pleural metastasis, Mini-thoracotomy, Testicular tumour spread

## Abstract

**Introduction:**

Testicular germ cell tumours metastasize in a very predictable fashion involving the retroperitoneal lymph nodes first followed by either lymphatic spread via thoracic duct or hematogenous spread to distant organs like lungs, liver and brain.

**Presentation of case:**

We encountered a case of 21-year-old gentleman with mixed germ cell tumour of testis who later presented with respiratory complaints which turned out to be a metastatic differentiated teratoma of unilateral pleura without involvement of retroperitoneal lymph nodes or any other organs.

**Discussion:**

Skip Metastasis to unilateral pleura is an extremely rare entity for testicular mixed germ cell tumour and no case has been reported in the literature so far.

## Highlights

• Mixed germ cell tumour with skip metastasis to unilateral pleura without involvement of any retroperitoneal lymph nodes or secondary to hematogenous spread involving mediastinal structures or any other distant sites is extremely rare.
• Histological subtype of metastatic lesion is different from the histological subtype of the primary tumour.
• Changing nature of histology of tumour with chemotherapy and progress of disease.
• Combined approach of chemotherapy and surgery is needed in case of mixed germ cell tumour depending on the histological subtype, metastasis and clinical presentation of the patient.

## 1 Introduction

Testicular neoplasms although are very rare, accounting for 1% of the malignancies in men. These tumours are the most common solid tumours in young adult men between 20 and 40 years of age. More than 90% of testicular neoplasms correspond to germ cell tumours that are divided into seminomatous and nonseminomatous tumours, corresponding to the histologic subtypes of embryonal carcinoma, yolk-sac tumour, teratoma and choriocarcinoma [1,2].

More than half of the germ cell tumours contain more than one cell type and are therefore known as mixed germ cell tumours [2].

The majority of the germ cell tumours metastasize to the lymph nodes in a characteristic pattern, although choriocarcinoma preferentially has hematogenous spread. Route of spread for testicular mixed germ cell tumour depends on the histological subtypes present in the tumour [2]. Retroperitoneal lymph nodes, particularly the para-aortic lymph-nodes on the left side, interaortocaval lymph nodes on the right side, are the primary and most common metastatic sites in the germ cell tumours [3].

The work has been reported in the line with the SCARE criteria [8].

## 2 Case report

21-year-old male with history of left sided testicular swelling with increased alfa fetoprotein levels (10,789 IU/ml) and beta-human chorionic gonadotropin levels (66.05 mIU/ml) with normal chest radiograph, underwent left high inguinal orchidectomy in December 2016 in the outside hospital. Histopathology report of the specimen was suggestive of “Mixed seminomatous and non seminomatous germ cell tumour with yolk sac tumour (40%), mature teratoma (55%) and seminoma (5%)”. Positron emitted tomography was done 1 month later post orchidectomy suggestive of “Diffuse metabolic activity seen in the left hemi-scrotum is secondary to recent surgery. There is no metabolically active lymph node, pleural, peritoneal, omental, solid visceral, osseous lesion, ascites or pleural effusion. Isolated non metabolic nodule in the left upper lobe and left lower lobe of the lung would need serial follow up”.

**Fig. 1:**
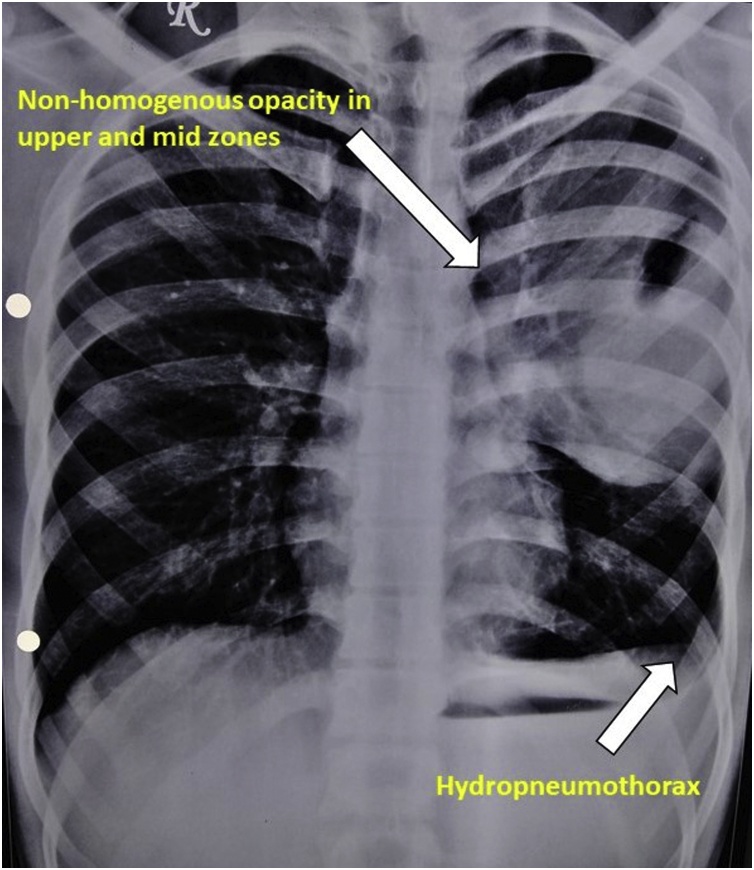
Chest radiography was done suggestive of “Non-homogenous opacity in the left upper and mid zones with left sided hydropneumothorax”.

Patient was asymptomatic for next 9 months and didn’t come for the regular follow-ups until he developed chest pain, breathlessness and severe back pain (Fig. 1).

Computed tomography of the chest done in September 2017 suggestive of “Area of collapse-consolidation in the periphery of apical-posterior segment of left upper lobe of lung and another well-defined oval radiopacity with sharp borders is noted in superior segment of lower lobe of left lung with left hydropneumothorax- nonspecific morphology probably of benign aetiology”.

Patient was managed with intercostal drainage tube insertion. Tumour markers were done showed Alpha fetoprotein levels (1606 IU/ml) and beta-human chorionic gonadotropin levels (5.40 mIU/ml). The pleural fluid sample was sent for cytology showed no malignant cells. subsequent radiographs were taken which confirmed the resolving pattern. Patient was discharged within 5 days after removal of the intercostal drainage tube.

Within 2 months patient again presented with severe respiratory complaints and repeat computed tomography of chest was done in December 2017 showed “subpleural nodule in left lower lobe of lung is suggestive of metastasis. This study is negative for any significant mediastinal adenopathy, interstitial lung disease or centrilobular opacities with shallow left hydro-pneumothorax”. Patient was again managed with intercostal drainage tube and pleural fluid sent for cytology was negative for any malignant cells.

**Fig. 2:**
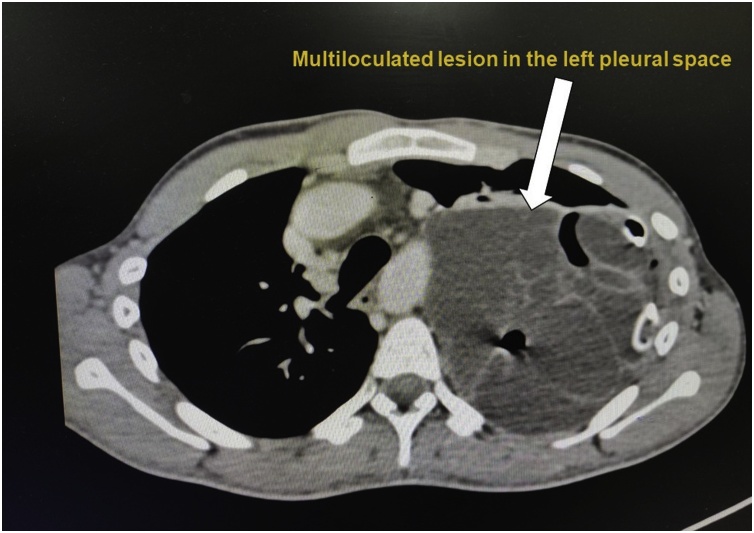
PET CT done in February 2018 showed “appearance of multiloculated lesions in left pleural space as a new finding”.

Patient was again hospitalised 2 times in 15 days with similar complaints for which pleurodesis was also attempted. But in view of recurrent pleural effusions which were not responding to medical management, patient was then subjected to 4 cycles of chemotherapy with Bleomycin, Etoposide and Cisplatin for a period of 2 months. Within a week after completion of chemotherapy patient was again admitted with severe respiratory complaints. The whole-body positron emitted tomography was repeated in February 2018 showed “Appearance of the multiloculated lesion in the pleural space on the left side is a new finding compared to the prior scan and should assumed to be metastatic unless proved otherwise with fluid aspiration cytology or biopsy of the solid component in a nodule. No retroperitoneal adenopathy seen” (Fig. 2).

**Fig. 3:**
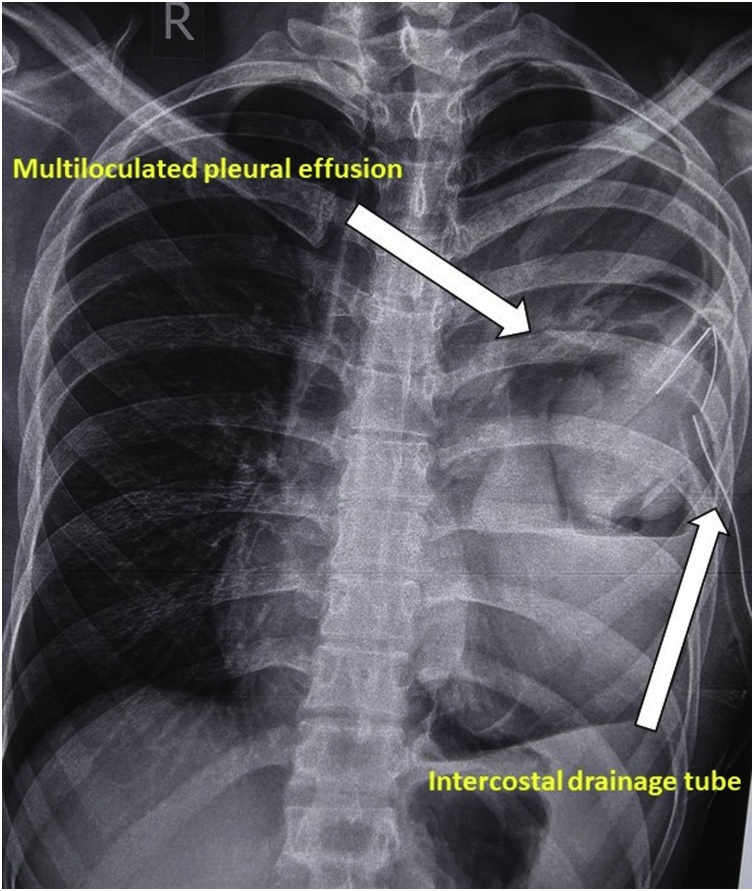
Chest radiograph showing multiloculated pleural effusion with intercostal drainage tube in situ.

Patient was subjected to salvage chemotherapy with Etoposide, Carboplatin and Ifosphamide in a period of March to May 2018. Pleural fluid aspiration under sonography guidance was done which again failed to show any malignant cells. (Fig. 3).

**Fig. 4:**
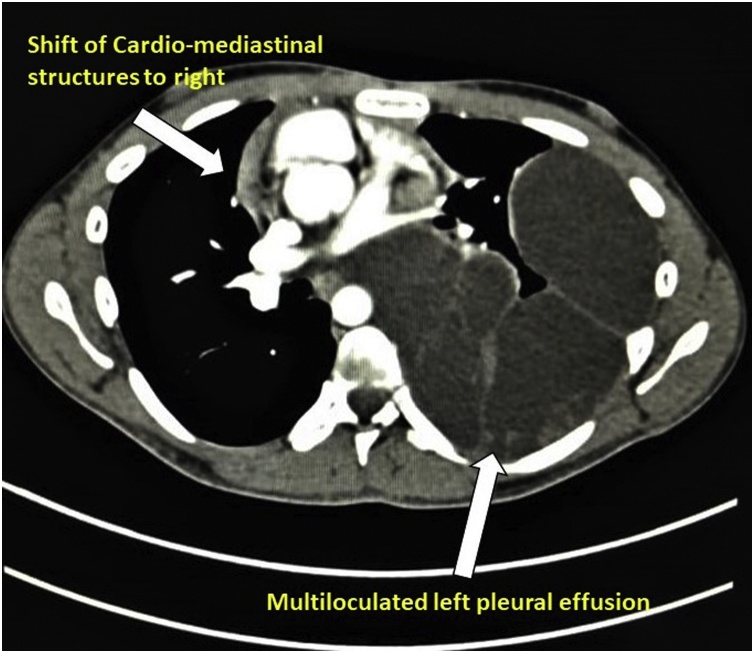
PET CT done post chemotherapy showed “considerable increase in cardiomediastinal structures with persistent multiloculated pleural effusion”.

Computed Tomography of chest at the end of 5^th^ chemotherapy cycle done, suggestive of “Considerable increased in shift of cardio-mediastinal structures. The rest of the morphological appearance of the loculated/septated left pleural effusion remains more or less unchanged with no significant mediastinal adenopathy or pulmonary metastasis.” (Fig. 4)

Patient was then referred to surgical team for further management. In view of worsening of the symptoms and sequential chest x-rays showing no improvement patient was subjected to “Left Mini-Thoracotomy with complete decortication of the diseased pleura” in July 2018. Histopathology report of this pleural sample was suggestive of “Differentiated teratoma- post chemotherapy pleural metastasis”.

**Fig. 5:**
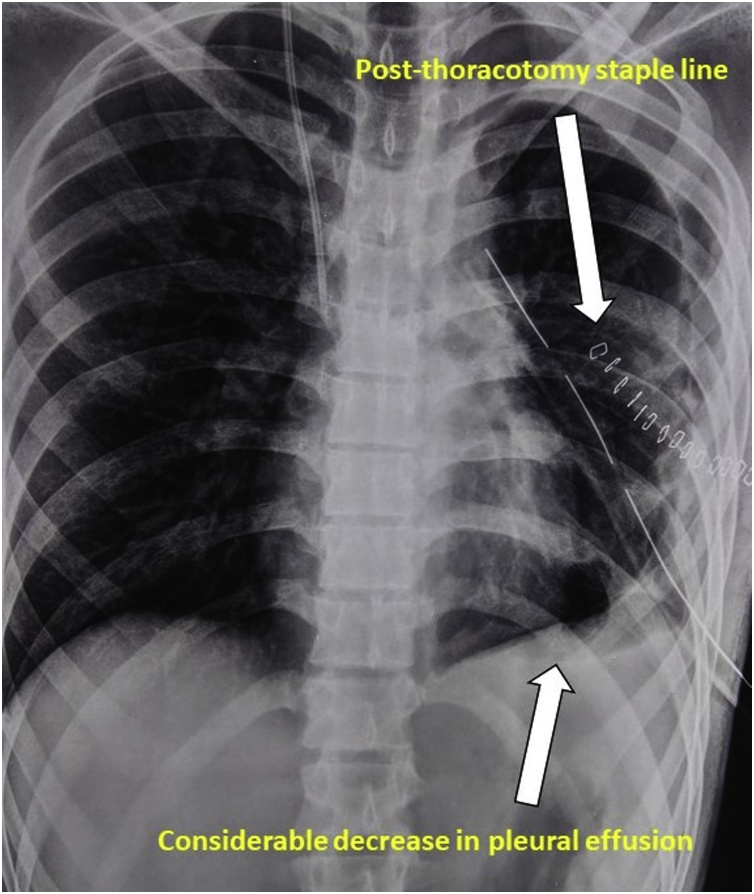
Chest radiography done post mini-thoracotomy showed "considerable decrease in pleural effusion”.

**Fig. 6:**
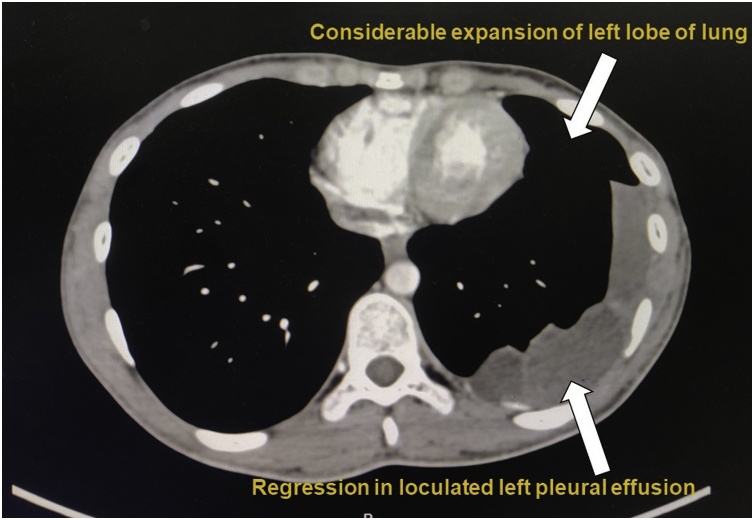
CT chest done 5 months post decortication of lung suggestive of “regression of left pleural effusion with considerable expansion of left lobe of lung”.

After the procedure sequential chest x-rays were taken each day which were showing resolving pattern of consolidation and pleural effusion with good lung expansion (Fig. 5). Patient is under regular follow up for last 9 months. Patient does not have any complaints, also is gaining weight, normal values of alfa fetoprotein levels (5.88 IU/ml) and beta-human chorionic gonadotropin levels (less than 2 mIU/ml). Computed tomography was done in December 2018 showed “Considerable regression in the loculated/septated left pleural effusion with considerable expansion of left lower lobe of lung and lingula compared to previous scan. No significant mediastinal adenopathy, pulmonary or pleural metastasis and no new lesion is seen” (Fig. 6).

## 3 Discussion

### 3.1 Classification

Testicular germ cell tumours are composed following cell types [2] (Table 1).

**Table 1:** Testicular germ cell tumour classification.

Mixed germ cell tumours occur in various combinations [1,6] (Table 2).

**Table 2:** Mixed germ cell tumour composition.

Serum elevation of alpha fetoprotein and beta human chorionic gonadotropin is common in mixed germ cell tumours, correlating with the presence of yolk sac tumour elements and syncytiotrophoblastic cells, respectively [2].

### 3.2 Pattern of spread

There appears to be a significant difference in the dissemination behaviour of metastasis between seminomas and nonseminomatous germ cell tumours at the pulmonary level, with latter being more frequent and more variable [4] (Table 3).

**Table 3:** Pattern of spread of mixed germ cell tumour.

Our patient was diagnosed with left testicular mixed germ cell tumour metastasizing to left pleura without involvement of any lymph nodes or distant organs which is very rare finding [5].

### 3.3 Change in histology of tumour

The pluripotential nature of germ cell tumours and, in particular, nonseminomatous tumours is evident from the varied histologic patterns of metastasis, more than half of which display different morphologies in primary versus metastatic sites, mainly due to chemotherapy and progress of disease [2]. As seen in the present case, primary tumour has yolk sac cells, seminoma cells and mature teratoma as a component, subsequent cytology from pleural aspirations were negative for malignant cells which has changed its morphology as differentiated teratoma in pleural metastasis [6].

### 3.4 Management

The decision on the mode of therapy largely relies on pathologic classification of germ cell tumours and spread of tumour. Between 65–85% of all seminomas are clinically confined to the testis, whereas 60–70% of non-seminomas present as recognizable metastatic disease [2]. Treatment of nonseminomatous or mixed germ cell tumours largely depends on whether the tumour is localized to the testis or has already metastasized to retroperitoneal lymph-nodes or other sites. Initial clinical and radiographic examination plays very important role in the management of the patient [1].

The established treatment of low-stage seminoma is inguinal orchidectomy followed by therapeutic or adjuvant radiation therapy. The optimal treatment of patients who present with distant metastases or bulky retroperitoneal disease is initial chemotherapy. The role of salvage chemotherapy, surgical removal, or radiation therapy for persistent radiographic masses remains controversial. Of major importance is the fact that roughly one third of patients with histologically pure seminoma of the testis who ultimately die of the disease are found to harbour nonseminomatous elements in metastatic sites [2,7].

Patients presenting with nonseminomatous germ cell tumours are subdivided into low, high and advanced stage disease. Patients with low stage may be candidates for surveillance, chemotherapy, or retroperitoneal lymph node dissection, depending on variety of factors such as the clinical staging, serum tumour markers, and tumour histologic findings. On the other hand, patients with advanced disease are further subcategorized into good and poor risk categories and are then subjected to primary and secondary chemotherapy depending on the nature of their disease [1,2,7].

## 4 Conclusion

1) Mixed germ cell tumour with skip metastasis to unilateral pleura without involvement of any retroperitoneal lymph nodes or secondary to hematogenous spread involving mediastinal structures or any other distant sites is extremely rare.
2) Histological subtype of metastatic lesion is different from the histological subtype of the primary tumour.
3) Changing nature of histology of tumour with chemotherapy and progress of disease is known.
4) Combined approach of chemotherapy and surgery is needed in case of mixed germ cell tumour depending on the histological subtype, metastasis and clinical presentation of the patient.

## Sources of funding

This research did not receive any specific grants from funding agencies in the public, commercial, or not-for-profit sectors.

## Ethical approval

This is a case report and does not require ethics committee approval.

## Consent

Written informed consent was obtained from the patient for publication of this case report and accompanying images. A copy of the written consent is available for review by the Editor-in-Chief of this journal on request.

## Author contribution

1) Dr Shravani Shetye: Conception and design, Acquisition of data, Analysis and interpretation of data, Drafting the article, Critical revision of the article, Final approval of the version to be published.

2) Dr Neerajpratap Singh: Conception and design, Acquisition of data, Analysis and interpretation of data. Drafting the article, Critical revision of the article, Final approval of the version to be published.

3) Manmohan Kamat: Conception and design, Acquisition of data, Analysis and interpretation of data.

4) Dr Katik Nattey: Drafting the article, Critical revision of the article.

5) Dr Seema Barman: Conception and design, Acquisition of data

## Registration of research studies

This is a case report and not a research study.

## Guarantor

Dr Manmohan Kamat.

## Provenance and peer review

Not commissioned, externally peer-reviewed.

## Declaration of Competing Interest

None declared.
